# Bibliographical Department

**Published:** 1880-07

**Authors:** 


### BIBLIOGRAPHICAL DEPARTMENT.

"Nothing extenuate, nor set down aught in malice."

"SURGERY IN THE PENNSYLVANIA HOSPITAL.—Being an epitome of the Practice in the Hospital since 1756. Including collations from the surgical notes, and an account of the more interesting cases from 1873 to 1878, with some statistical tables, by Thomas G. Morton, M. D. and William Hunt, M. D., surgeons to the hospital; with papers by John B. Roberts, M. D. and Frank Woodberry, M. D., late resident physicians in the hospital. Prepared by direction of the managers of the hospital, Philadelphia, J. B. Lippincott & Co., London; 16 Southampton street, Covent Garden, 1880."

We are indebted to the courtesy of the senior surgeon, Dr. Thomas George Morton, for a copy of the above book, and after a pretty thorough examination of its contents have no hesitation in designating it as the most valuable contribution to surgery, so far as it extends, of which we have any acquaintance or knowledge. The views, opinions and operations, worded upon its pages, are judicious, practical and thoroughly orthodox in every respect, in the department of the profession to which it belongs. It is refreshing in this day "of book-making of which there seems to be no end," to obtain possession of so *practical* a work. We took brief notes on several of the subjects contained in it, as we turned over its pages intending to lay them before our readers; but we find it impracticable to do so now, in consequence of want of space. Most of the matter contained in the volume is from the pen of Dr. Morton, and we feel sure that all who may be so fortunate as to secure a copy of the work will agree with us in according to himself and colleagues, the plaudit of "well done good and faithful servants," when they have risen from a careful perusal of the "Surgery of the Pennsylvania Hospital."

Homœopathy; what is it? A statement and Review of Its Doctrines and Practice, by A. B. Palmer, A. M., M. D., Professor of Pathology and Practice of Medicine in the College of Medicine and Surgery in the University of Michigan, etc., Detroit; George S. Davis, Medical Publisher, 1880." We are in receipt of a copy of the above work and beg to extend our thanks to the publisher for the same. Though familiar with the leading parts contained in the book for several years past, we have read the author's statements of them with renewed pleasure and satisfaction. We wish the book was in the hands of *all* our Homœopathic acquaintances and friends, professional and lay, as we feel sure they would be *profited* by its perusal though they should be so through spectacles deeply shaded by prejudice. It is a book very well suited for a place on the centre table of, the office of the regular physician, and could not fail to be of great use to intelligent persons generally.

Mid-Continent Presbyterian, published at Kansas City, Mo., comes regularly to our sanctum. It is a well-edited and valuable religious newspaper, of liberal and enlightened views upon theological matters, and is consequently worthy the patronage of the christian public.

The Hahnemannian Monthly, published by the Hahnemanne Club of Philadelphia, published monthly at three dollars per annum. As its title imports, the above journal is a medium of communication between the gentlemen who profess to administer medicines in *infinitesimal* quantities, and to practice under the banner of *Similia Similibus Curantur.* For persons of this belief, we have no doubt it is a serial of much importance, as it is very neatly gotten up. Those, not of this faith, who would like to see a little of the *interior* of the Homœopathic system might find something to interest them in some of its articles. It seems to be conducted with commendable fairness, and as such we willingly enter it upon our exchange list.

A Contribution to the Pathology of the Cicatrices of Pregnancy, by Samuel C. Busey, M. D., Professor of the Theory and Practice of

Medicine, Medical Department University of Georgetown, D. C., etc., is an interesting reprint from the Gynecological Transactions.

Announcement of the Medical Department of the University of Pennsylvania for the 115th Annual Session, 1880-81, shows this venerable and time honored institution to be in a healthful and vigorous condition.

We are indebted to Dr. W. H. Geddings for a pamphlet copy of "Bethlehem, (New Hampshire,) as a resort for Health and Pleasure." Dr. Gedding's spends his summer in that salubrious village, and those who may find their way thither in quest of health could not do better than consult him.

